# Neurologic complications of influenza requiring pediatric intensive care: a multicenter study

**DOI:** 10.62675/2965-2774.20260173

**Published:** 2026-09-25

**Authors:** Nihal Akçay, İlyas Bingöl, Demet Tosun, Muhterem Duyu, Leyla Telhan, Özge Umur, Güntülü Şık, Mehmet Emin Menentoğlu, Özel Abdulrahman, Ceren Bilgün, Emrullah Aygüler, Yüce Servet

**Affiliations:** 1 Department of Pediatric Intensive Care Unit University of Health Sciences Kanuni Sultan Suleyman Training and Research Hospital Istanbul Turkey Department of Pediatric Intensive Care Unit, University of Health Sciences Kanuni Sultan Suleyman Training and Research Hospital - Istanbul, Turkey.; 2 Department of Pediatric Intensive Care Unit Istanbul Medeniyet University Göztepe Prof. Dr. Süleyman Yalçın City Hospital Istanbul Turkey Department of Pediatric Intensive Care Unit, Istanbul Medeniyet University, Göztepe Prof. Dr. Süleyman Yalçın City Hospital - Istanbul, Turkey.; 3 Department of Pediatric Intensive Care Unit Istanbul Medipol University Istanbul Turkey Department of Pediatric Intensive Care Unit, Istanbul Medipol University - Istanbul, Turkey.; 4 Department of Pediatric Intensive Care Unit Istanbul Acıbadem University Istanbul Turkey Department of Pediatric Intensive Care Unit, Istanbul Acıbadem University - Istanbul, Turkey.; 5 Department of Pediatric Intensive Care Unit Faculty of Medicine Istanbul Cerrahpaşa University Istanbul Turkey Department of Pediatric Intensive Care Unit, Faculty of Medicine, Istanbul Cerrahpaşa University - Istanbul, Turkey.; 6 Department of Pediatric Intensive Care Unit University of Health Sciences Bakırköy Dr. Sadi Konuk Training and Research Hospital Istanbul Turkey Department of Pediatric Intensive Care Unit, University of Health Sciences Bakırköy Dr. Sadi Konuk Training and Research Hospital - Istanbul, Turkey.; 7 Department of Pediatric Intensive Care Unit University of Health Sciences Bağcılar Training and Research Hospital Istanbul Turkey Department of Pediatric Intensive Care Unit, University of Health Sciences Bağcılar Training and Research Hospital - Istanbul, Turkey.; 8 Department of Pediatric Intensive Care Unit University of Health Sciences Ümraniye Training and Research Hospital Istanbul Turkey Department of Pediatric Intensive Care Unit, University of Health Sciences Ümraniye Training and Research Hospital - Istanbul, Turkey.; 9 Department of Pediatric Intensive Care Unit Faculty of Medicine Istanbul University Istanbul Turkey Department of Pediatric Intensive Care Unit, Faculty of Medicine, Istanbul University - Istanbul, Turkey.; 10 Department of Public Health Faculty of Medicine Istanbul University Istanbul Turkey Department of Public Health, Faculty of Medicine, Istanbul University - Istanbul, Turkey.

## INTRODUCTION

Influenza is a common viral infection associated primarily with respiratory symptoms; however, neurologic complications may occur in a subset of pediatric patients and can lead to severe morbidity and mortality. Reported neurologic manifestations include febrile seizures, status epilepticus, encephalopathy, encephalitis, acute necrotizing encephalopathy, acute disseminated encephalomyelitis, stroke, and other neuroinflammatory syndromes.^( 1 , 2 )^

Although most children with influenza recover uneventfully, severe neurologic involvement may require admission to pediatric intensive care units (ICUs). Recent reports have highlighted increasing recognition of influenza-associated encephalopathy and acute necrotizing encephalopathy in children, particularly during the post-COVID-19 period.^( 3 , 4 )^However, multicenter data describing critically ill pediatric patients with influenza-associated neurologic complications remain limited.

In this multicenter study, we aimed to describe the clinical characteristics, organ dysfunction patterns, management strategies, and short-term outcomes of children admitted to pediatric ICUs with influenza-associated neurologic complications and to explore potential differences between influenza A and influenza B infections.

## METHODS

This multicenter retrospective study included children aged 1 month to 18 years with laboratory-confirmed influenza and neurologic complications admitted to nine pediatric ICUs in Istanbul, Turkey, between July 2022 and April 2025. We confirmed influenza infection by polymerase chain reaction testing. We defined neurologic complications as seizures, encephalopathy, or encephalitis occurring within 5 days of influenza-like illness onset without an alternative etiology. We defined encephalopathy as altered mental status lasting ≥ 24 hours, and encephalitis as encephalopathy accompanied by at least two of the following: fever, focal neurologic findings, cerebrospinal fluid pleocytosis, electroencephalographic abnormalities, or neuroimaging findings suggestive of inflammation or infection. We included only children with influenza-associated neurologic complications aged 1 month to 18 years. Therefore, we excluded patients without neurologic involvement and neonates.

We retrospectively collected clinical, laboratory, treatment, and outcome data from medical records using standardized case report forms. Organ dysfunctions were defined according to standardized pediatric critical care criteria, including Kidney Disease: Improving Global Outcomes (KDIGO) criteria for renal failure and vasoactive support requirement for hemodynamic failure.^( 5 )^Illness severity was assessed using the Pediatric Risk of Mortality III (PRISM III) score^( 6 )^ and Pediatric Glasgow Coma Scale (pGCS)^( 7 )^within the first 24 hours of pediatric ICU admission.

Categorical variables were compared using Pearson’s Chi-squared or Fisher’s exact test, while continuous variables were analyzed using the independent samples t-test or Mann-Whitney U test, as appropriate. A two-sided p value < 0.05 was considered statistically significant. Statistical analyses were performed using IBM SPSS Statistics version 30.0 (IBM Corp., Armonk, NY, USA).

The local Ethics Committee approved the study (KAEK/2025.02.35), and the study was conducted in accordance with the Declaration of Helsinki. Because this was a retrospective study, the Ethics Committee waived the requirement for informed consent.

## RESULTS

A total of 44 children with laboratory-confirmed influenza-associated neurologic complications were included, including 32 influenza A and 12 influenza B cases. Median age was 4.13 years (interquartile range [IQR] 1.62 - 7.82), and 65.9% were male. Influenza vaccination rates were low in both groups (6.8% overall).

Seizures (86.4%) and altered consciousness (70.5%) were the most common neurologic manifestations. Reasons for pediatric ICU admission differed between groups (p = 0.014): encephalopathy and encephalitis were more frequent among influenza A patients, whereas sepsis/multiorgan dysfunction syndrome and pneumonia/acute respiratory distress syndrome were more common in Influenza B cases. Mortality was 4.5% overall.

All patients received oseltamivir. Influenza B patients more frequently received corticosteroids, therapeutic plasma exchange, and inotropic support. Renal failure (41.7% *versus* 3.1%; p < 0.001) and hemodynamic failure (41.7% *versus* 12.9%; p = 0.038) were also more common in Influenza B cases.

Magnetic resonance imaging abnormalities were detected in 43.2% of patients, including restricted diffusion, hemorrhagic lesions, and necrotic changes. Acute necrotizing encephalopathy and acute disseminated encephalomyelitis were observed only in Influenza A patients. Electroencephalographic abnormalities were present in 29.5% of cases ( Table 1 ).

**Table 1:** Clinical characteristics, organ dysfunction, and outcomes of children with influenza-associated neurologic complications

Parameter	Influenza A (n = 32)	Influenza B (n = 12)	Total (n = 44)	p value
Age (years)	3.77 (1.65 - 8.56)	5.36 (1.46 - 7.57)	4.13 (1.62 - 7.82)	0.990
Male sex	19 (59.4)	10 (83.3)	29 (65.9)	0.127
Influenza vaccination	2 (6.3)	1 (8.3)	3 (6.8)	0.807
Convulsions	29 (90.6)	9 (75.0)	38 (86.4)	0.179
Altered consciousness	20 (62.5)	11 (91.7)	31 (70.5)	0.059
Primary reason for pediatric ICU admission				
Encephalopathy	11 (34.4)	0 (0.0)	11 (25.0)	0.014*
Status epilepticus	7 (21.9)	3 (25.0)	10 (22.7)	
Sepsis/MODS	1 (3.1)	4 (33.3)	5 (11.4)	
Others	13 (40.6)	5 (41.7)	18 (40.9)	
Renal failure	1 (3.1)	5 (41.7)	6 (13.6)	< 0.001*
Hemodynamic failure	4 (12.9)	5 (41.7)	9 (20.9)	0.038*
MRI abnormalities	14 (43.8)	5 (41.7)	19 (43.2)	0.897
EEG abnormalities	11 (34.4)	2 (16.7)	13 (29.5)	0.187
Neurologic/behavioral sequelae at discharge	13 (40.6)	6 (50.0)	19 (43.2)	0.113
Mortality	1 (3.1)	1 (8.3)	2 (4.5)	0.460

ICU - intensive care unit; MODS - multiorgan dysfunction syndrome; MRI - magnetic resonance imaging; EEG - electroencephalography. We used the Mann-Whitney U test and Pearson Chi-squared test as appropriate. * Statistical significance. Results expressed as mean (interquartile range) or n (%).

## DISCUSSION

In this multicenter cohort of children admitted to pediatric intensive care units with influenza-associated neurologic complications, seizures, encephalopathy, and altered consciousness were the predominant clinical manifestations. Despite relatively low overall mortality (4.5%), nearly one-third of survivors experienced neurologic or behavioral sequelae at hospital discharge, underscoring the substantial morbidity associated with severe neuroinfluenza. Similar rates of persistent neurologic impairment have been reported in previous multicenter pediatric studies of influenza-associated encephalopathy.^( 8 )^

Recent surveillance reports from the Centers for Disease Control and Prevention have highlighted increasing recognition of influenza-associated encephalopathy and acute necrotizing encephalopathy in children during recent influenza seasons.^( 9 , 10 )^In our cohort, acute necrotizing encephalopathy was observed exclusively in influenza A patients, whereas influenza B cases more commonly demonstrated systemic and multiorgan involvement, including renal and hemodynamic dysfunction. However, these findings should be interpreted cautiously given the limited number of influenza B cases and the descriptive nature of the analysis.

Our findings are consistent with previous reports demonstrating that severe neurologic involvement in pediatric influenza is frequently associated with critical illness and organ dysfunction.^( 11 - 13 )^The high frequency of neurologic sequelae observed in our study further emphasizes the need for early recognition and close neurologic follow-up in affected children.

Comparisons between influenza A and influenza B subgroups should be considered exploratory and hypothesis-generating because of the limited sample size and multiple comparisons.

Several limitations should be acknowledged, including the retrospective design, relatively small sample size, lack of standardized long-term neurologic follow-up, and potential heterogeneity in treatment approaches across participating centers. In addition, the absence of a control group precludes causal inference and limits interpretation of differences between influenza A and B infections.

Despite these limitations, this study represents one of the largest multicenter pediatric ICU cohorts of pediatric influenza-associated neurologic complications reported from Turkey. Larger prospective studies are needed to better define prognostic factors and long-term neurologic outcomes in critically ill children with neuroinfluenza.

### Ethics approval

This multicenter retrospective study was approved by the Ethics Committee of the University of Health Sciences, Kanuni Sultan Süleyman Training and Research Hospital, Istanbul, Turkey (approval No: KAEK/2025.02.35). We obtained institutional permissions for data use from all participating centers. Because of the study’s retrospective design, the Ethics Committee waived the requirement for written informed consent. The study was conducted in accordance with the ethical principles of the Declaration of Helsinki.
